# Supplementing claims data with outpatient laboratory test results to improve confounding adjustment in effectiveness studies of lipid-lowering treatments

**DOI:** 10.1186/1471-2288-12-180

**Published:** 2012-11-26

**Authors:** Sebastian Schneeweiss, Jeremy A Rassen, Robert J Glynn, Jessica Myers, Gregory W Daniel, Joseph Singer, Daniel H Solomon, SeoYoung Kim, Kenneth J Rothman, Jun Liu, Jerry Avorn

**Affiliations:** 1 Division of Pharmacoepidemiology and Pharmacoeconomics, Department of Medicine, Brigham and Women’s Hospital, 1 Brigham Circle, Suite 3030, Boston, 02120, MA, USA; 2 The Brookings Institution, Washington, DC, USA; 3 HealthCore Inc, Wilmington, DE, USA; 4 RTI Health Solutions, RTP, Durham, NC, USA

**Keywords:** Insurance claims data, Laboratory test results, Serum lipid levels, Confounding, Imputation, Pharmacoepidemiology, Lipid lowering therapy, Statin, Ezetimibe

## Abstract

**Background:**

Adjusting for laboratory test results may result in better confounding control when added to administrative claims data in the study of treatment effects. However, missing values can arise through several mechanisms.

**Methods:**

We studied the relationship between availability of outpatient lab test results, lab values, and patient and system characteristics in a large healthcare database using LDL, HDL, and Hb_A1c_ in a cohort of initiators of statins or Vytorin (ezetimibe & simvastatin) as examples.

**Results:**

Among 703,484 patients 68% had at least one lab test performed in the 6 months before treatment. Performing an LDL test was negatively associated with several patient characteristics, including recent hospitalization (OR = 0.32, 95% CI: 0.29-0.34), MI (OR = 0.77, 95% CI: 0.69-0.85), or carotid revascularization (OR = 0.37, 95% CI: 0.25-0.53). Patient demographics, diagnoses, and procedures predicted well who would have a lab test performed (AUC = 0.89 to 0.93). Among those with test results available claims data explained only 14% of variation.

**Conclusions:**

In a claims database linked with outpatient lab test results, we found that lab tests are performed selectively corresponding to current treatment guidelines. Poor ability to predict lab values and the high proportion of missingness reduces the added value of lab tests for effectiveness research in this setting.

## Background

Administrative health insurance claims databases provide comprehensive and longitudinal records of encounters with the health care system and of drug dispensing, but lack clinical detail. For example, while the performance of a lab test will generate a claim, the test result will not be available within the claims database. This shortcoming can be overcome by merging outpatient laboratory test results extracted from electronic medical records (EMR) systems with claims data. Adjusting for lab results may result in better confounding control when administrative claims data are used to study treatment effects of medical products.

A difficulty arises from the way in which lab tests are ordered and performed in the American health care system. EMR systems with outpatient lab results generally rely on major laboratory companies to supply lab results data; results for patients whose tests are conducted outside the large chains may go unrecorded in EMRs. In conducting comparative effectiveness research in claims data, pharmacoepidemiologists generally interpret the absence of a claim as the absence of that service/diagnosis, which can result in a covariate misclassification problem but not a missing data problem [1]. However, missing lab test results do not mean the test was not performed or the results are normal, and thus must be handled like other missing data. There is little guidance in the literature on the nature of the missingness of such laboratory information or whether missing lab test results can be adequately imputed.

Investigators have employed varying strategies to deal with this situation. One approach is to identify a subcohort of patients with complete information on the lab test results of interest. Seeger et al. studied the effectiveness of statin therapy to reduce myocardial infarction rates, by requiring all patients studied to have a recorded LDL > 130 mg/dl [2]. This approach reduces the proportion of subjects with missing data, but that advantage comes at the cost of fewer subjects in the study, and a final study population that may be dissimilar to the broader patient population for important characteristics. Furthermore, this approach is impractical for multiple unrelated lab test results, as complete cases may be few [3].

**Figure 1:**
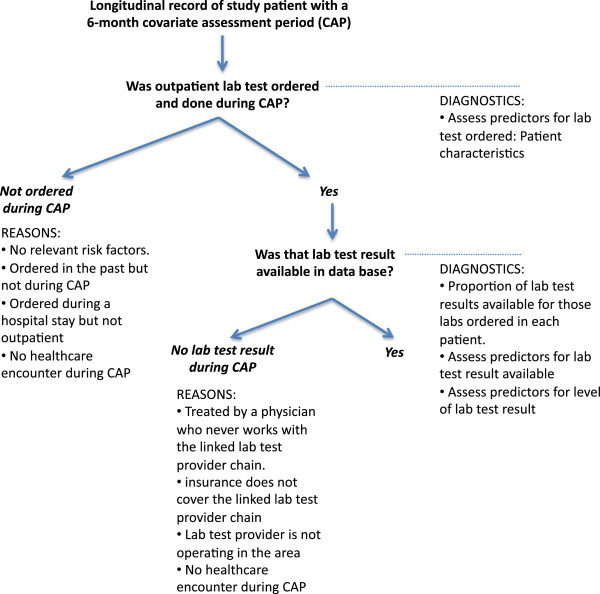
Reasons for missing lab test results in a longitudinal healthcare utilization database linked to a lab test provider database*. * In the setting of a new user cohort study with a defined covariate assessment period before the first exposure and before follow-up

One unfavorable approach is to include an indicator term for missing lab test data. In the case of lab results, missingness can imply three things: (1) the physician did not see a need to have a test ordered; (2) the patient did not choose to have the test performed, or (3) the test was performed but not in a facility whose data fed back to the patient’s EMR (Figure 1). Though the implication of each of these cases is entirely different, they are indistinguishable in the data; as such, coding them simply as “missing” would lead to bias. Even if all the missingness were due to the third case, in which data are most plausibly missing completely at random, the use of a missing indicator term could still cause bias [4,5].

In order to further meaningful comparative effectiveness research, we must understand the selectiveness of missing lab test results and how missingness may be related to study outcomes. In this paper, we seek to describe the analytic issues encountered when lab results may not be available for many patients. As an example, we describe the characteristics associated with the absence of laboratory test results and the degree to which missingness and actual lab test values can be predicted based on patient and health plan characteristics in a population of patients initiating lipid-lowering therapy.

## Methods

Database studies that combine claims and lab test results or other data from EMRs typically employ the claims as a “data backbone,” as claims data provide a longitudinal view of virtually all health care encounters and drug dispensings submitted for health insurance reimbursement. Increasingly, claims databases link data from large national lab test chains [6-8]. Though the chains service a large number of American patients, the resulting linked data may cover substantially less than 50% of outpatient lab tests, with coverage highly dependent on the region where the patient resides and the lab companies servicing that region. Figure 1 illustrates two levels of missingness that may arise in such situations. No claim will be recorded (Level 1) if a physician does not order a test, a patient receives a lab in a hospital, or a patient does not get a test that was ordered. The result of a test that was performed may not be transmitted to the patient’s claims data (Level 2) if the insurer has not established a data exchange agreement with the laboratory provider. The likelihood of Level 2 missingness increases if there is no laboratory provider operating in the area that has a data exchange agreement with the insurer.

### Data sources

We employed longitudinal claims data from 14 Blue Cross and/or Blue Shield-licensed health plans of Wellpoint across 14 US states, as represented in the HealthCore Integrated Research Database^SM^ (HIRD^SM^). HealthCore linked claims information to lab test results provided by two large national laboratory providers, for laboratory tests performed between January 1, 2005 through June 30, 2010 on patients represented in the HIRD system. The claims data contained information on drug dispensings, outpatient medical services, and hospitalizations including emergency room visits. All medical services were coded with up to 9 discharge diagnoses [1]. Individual laboratory test results were identified by LOINC codes and standardized across lab providers. This study was approved by the Brigham and Women’s Hospital Institutional Review Board and signed data use agreements were in place.

### Study cohort and exposure

**Figure 2:**
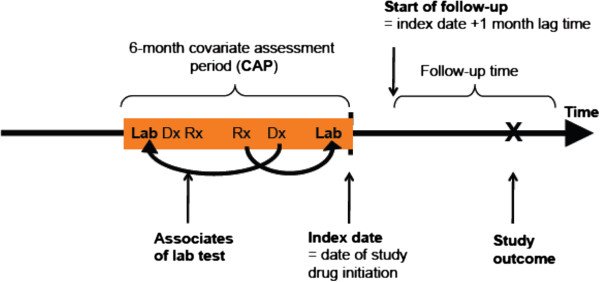
Incident user cohort study*. * The 6-month covariate assessment period (CAP) precedes the initiation of treatment. During the CAP we identified patient characteristics, including lab tests performed and lab test results available. After treatment start followed a 1-month lag period before events were attributed to the treatment. The arrows between prescriptions (Rx), diagnoses (Dx) and lab tests denote the fact that the temporality of events within the CAP was not considered in this study

From the data available, we established a cohort of all incident users of any statin (simvastatin, pravastatin, lovastatin, atorvastatin, rosuvastatin), Vytorin (simvastatin plus ezetimibe), or ezetimibe who were 18 years or older at the start of treatment. Incident use was established by requiring at least 12 months of insurance coverage before treatment and no use of any lipid-lowering therapy in those 12 months. 1 All covariate information was assessed in the longitudinal healthcare claims over a covariate assessment period (CAP) starting 6 months before treatment initiation and up to the day of dispensing of the index drug. Follow-up for occurrence of MI started 1 month after initiation of lipid-lowering treatment, a conservative assumption to allow for the biologic action of the medication to occur (Figure 2) [9]. We categorized each medication on the index date into high and low intensity treatment based on its ability to lower LDL levels (Table 1) [10].

**Table 1:** Definitions for high vs. low intensity lipid-lowering therapy

**Generic entity of study medications**	**Brand name**	**High-intensity daily doses (mg)**	**Low-intensity daily doses (mg)**
Atorvastatin	Lipitor	> 10	≤ 10
Fluvastatin	Lescol	All doses considered low-intensity	
Lovastatin	Mevacor	> 40	≤ 40
Pravastatin	Pravachol	All doses considered low-intensity	
Rosuvastatin	Crestor	> 5	≤ 5
Simvastatin	Zocor	> 40	≤ 40
Ezetimibe	Zetia		
Ezetimibe + simvastatin fixed combination	Vytorin	If simvastatin component > 40	If simvastatin component ≤ 40

We defined two subgroups of patients with chronic conditions. Rheumatoid arthritis (RA) was defined as at least two outpatient diagnoses of RA in the CAP or one hospital discharge diagnosis of RA in CAP or one diagnosis of RA plus dispensing of a disease modifying anti-rheumatic drug. Diabetes (DM) was defined as at least two outpatient diagnoses of DM in the CAP or one hospital discharge diagnosis of DM in CAP or one diagnosis of DM plus an insulin or oral antidiabetic dispensing. Patients with rheumatoid arthritis and diabetes were identified as subgroups with chronic conditions because these patients were likely to receive more lab tests at regular intervals than the typical patient initiating a statin. Patients with rheumatoid arthritis are of further interest in that they may receive care primarily from a specialist rather than an internist and therefore, may have different patterns of laboratory use.

### Patient characteristics and lab test results

Patient characteristics and potential confounders assessed during the 6-month CAP included age (18–40; 41–64; 65+), sex, state of residence, insurance plan type (Health Maintenance Organization, Medicare Advantage, Medicare Supplemental, Preferred Provider Organizations, Indemnity, other), number of physician visits, number of cardiologist visits, number of different drugs used, [11] hospitalization in the 30 days prior to treatment initiation, hospitalization for more than 30 days before treatment initiation, number of days hospitalized, number of outpatient lab test ordered, hypercholesterolemia, hypertension, heart failure, myocardial infarction, coronary revascularization, peripheral vascular disease, peripheral arterial revascularization, TIA/stroke, carotid revascularization, pre-diabetes, diabetes, arthritis, COPD, oxygen canister use, and obesity. Clinical covariates were assessed based on the presence of ICD-9 diagnosis codes (see Additional file 1: Appendix Table S1) in administrative claims during the CAP. In this exploratory analysis, we included a wide range of clinical covariates frequently measured in claims-based studies.

Within the 6 months covariate assessment period we identified all recorded outpatient lab test results for 23 commonly-performed lab tests, including lipid tests, Hb_A1c_, and others (see Additional file 1: Appendix Table S2). Additionally, we used CPT-4 codes to identify all labs for which charges were claimed during the CAP. We chose to include 23 lab tests to increase the probability that patients would have multiple lab tests performed and that we would be able to asses whether lab values were missing at the patient level or the test level.

In comparative effectiveness research, as in other areas of clinical epidemiology, missing data are both common and problematic. Imputation of missing values may increase precision and validity of effect estimates. The imputation literature recommends including not only pre-exposure patient characteristics and treatment information in the prediction of missing values but also information on the outcome status [12]. In our example, outcomes of interest were the incidence of myocardial infarction (assessed with a positive predictive value of 94%); [13] hospitalization for acute coronary syndrome (ACS) that included a coronary revascularization procedure; stroke; and death attributed to any cause (see Additional file 1: Appendix Table S3). Follow-up time started 1 month after initiation of a cholesterol-lowering drug (Figure 2). Patients were censored at the time of discontinuation of the index drug, any of the outcomes, disenrollment, or study end (June 30, 2010), whichever came first.

### Analysis

In this analysis, ascertainment of performing a lab test refers only to tests performed in the outpatient setting. We determined the proportion of patients who had at least one such lab test performed out of the 23 study lab tests and then focused on 3 specific cardiovascular risk markers: LDL, HDL, and Hb_A1c_[14]. In sensitivity analyses, we extended the 6-month covariate assessment period to 9 and to 12 months in an effort to capture more lab test results.

In order to quantify differential lab test performance and result availability, we computed the number of lab tests performed (as measured by the presence of CPT-4 codes) and the proportion of those with test results available in the linked database. We then cross-tabulated these data with patient and system characteristics.

For each of the LDL, HDL, and Hb_A1c_ cardiovascular disease risk markers, any factors associated with a completed test were identified in a multivariate logistic regression that predicted whether the outpatient lab test was performed, as a function of the patient and system characteristics described above plus statin/Vytorin exposure and cardiovascular outcome status. We then determined overall sensitivity and specificity for the predicted probabilities of test performance and model c-statistics.

In order to explore the performance of imputation strategies, we fit linear regression models for the patients who had lab test results available, in order to predict the actual LDL, HDL, and Hb_A1c_. In instances where patients received multiple tests, we used the value from the last test. We assumed normal distributions of test results as reasonable approximations, although data were slightly skewed. We express the proportion of explained variation as the observed R^2^ from the linear regression models.

Lastly, we investigated the relationship between the completion of a lab test, the availability of test results in our database, and whether the test results themselves differed between study exposure groups stratified by RA and diabetes.

## Results

Over the study period we identified 703,484 patients who met the study eligibility criteria and initiated lipid-lowering therapy with statins, ezetimibe, or a combination of both. Among those, 68% had a recorded charge for at least one of the 23 study lab tests in the 6 months before treatment (Table 2). This proportion increased to 72% if the covariate assessment period was extended to 9 months before treatment, and to 74% during a 12-month period. For patients with diabetes or RA the proportions were higher (80% during 6 months) but showed equally small increases if the covariate assessment period was extended (83% and 84%). For LDL and Hb_A1c_ tests, the proportion of patients with a recorded charge for at least one test during the 6 months before initiation of lipid-lowering therapy was about 60% and 17%, respectively. For patients with diagnosed diabetes, 68% had a charge for an Hb_A1c_ test (Table 2).

**Table 2:** Number of patients with at least one lab test performed and claimed among 703,484 initiators of statins and/or ezetimibe using 6, 9, and 12-month confounder assessment periods (CAPs)

	**All patients**	Number (%) of patients with at least 1 lab test performed*						**Number (%) of patients with at least 1 LDL lab test performed**						**Number (%) of patients with at least 1 LDL lab test performed**					
		**6 month CAP**		**9 month CAP**		**12 month CAP**		**6 month CAP**		**9 month CAP**		**12 month CAP**		**6 month CAP**		**9 month CAP**		**12 month CAP**	
		**N**	**%**	**N**	**%**	**N**	**%**	**N**	**%**	**N**	**%**	**N**	**%**	**N**	**%**	**N**	**%**	**N**	**%**
All patients	703,484	481,133	68%	505,161	72%	520,559	74%	421,708	60%	445,205	63%	460,293	65%	121,764	17%	132,358	19%	139,784	20%
DM patients	111,684	89,344	80%	92,185	83%	93,952	84%	74,576	67%	78,598	70%	81,104	73%	76,413	68%	79,932	72%	81,965	73%
RA patients	4,523	3,676	81%	3,774	83%	3,840	85%	2,650	59%	2,813	62%	2,915	64%	839	19%	918	20%	984	22%

* All tests = any of a set of 23 different frequently-performed laboratory tests recorded in study database.

Overall and regardless of having a test performed, the proportion of patients with any outpatient lab test results available in the linked database was about 30%, which was similar in patients with diabetes or RA. Lab test results for LDL or HDL were available for about 20% of patients during the 6 months before initiation of lipid-lowering therapy.

Table 3 shows whether any of 23 outpatient lab tests, including LDL, HDL and Hb_A1c_ were performed within the 6 months before initiating lipid-lowering therapy cross-tabulated by a range of patient and health system characteristics. Overall, 481,133 (68%) of study patients had claims evidence of an outpatient lab test and 42% thereof had results available in the study data (29% of all patients). The proportion with at least one lab test performed varied substantially by patient characteristics, while test result availability varied little, and only for variables such as system characteristics and state of residence (Table 3).

**Table 3:** Patients with lab test results reported in study population of 703,484 initiators of statins or ezetimibe using a 6-month covariate assessment period*

**Patient characteristics as determined in CAP**	**All patients**	**Patients with at least 1 of any 23 lab tests**^**1)**^					**Patients with at least 1 LDL test**					**Patients with at least 1 HDL test**					**Patients with at least 1 Hb**_**A1c**_**test**				
		**Tests performed**		**Results available**			**Tests performed**		**Results available**			**Tests performed**		**Results available**			**Tests performed**		**Results available**		
	**N**	**N**	**% of total**	**N**	**% of total**	**% of done**	**N**	**%**	**N**	**%**	**% of performed**	**N**	**%**	**N**	**%**	**% of performed**	**N**	**%**	**N**	**%**	**% of performed**
All patients	703,484	481,133	68	204,143	29	42	421,708	60	178,254	25	42	417,598	59	176,780	25	42	121,764	17	56,653	8	47
Age																					
18-40	71,396	52,674	74	23,861	33	45	48,565	68	21,313	30	44	48,021	67	21,150	30	44	12,424	17	5,770	8	46
41-64	489,581	363,406	74	150,096	31	41	329,097	67	131,999	27	40	325,945	67	130,912	27	40	92,076	19	40,635	8	44
65+	142,507	65,053	46	30,186	21	46	44,046	31	24,942	18	57	43,632	31	24,718	17	57	17,264	12	10,248	7	59
Male sex	378,177	256,537	68	107,876	29	42	225,308	60	94,715	25	42	223,007	59	93,962	25	42	66,202	18	30,286	8	46
# of days hospitalized	1	1		1			0	54	0	51	93	0	55	0	51	91	1	91	1	78	86
Hospitalized in 30 days before treatment initiation (y/n)	52,959	29,933	57	8,871	17	30	15,942	30	5,738	11	36	15,929	30	5,691	11	36	6,353	12	2,483	5	39
Hospitalized in 31 to 180 days before treatment initiation (y/n)	39,304	28,823	73	10,569	27	37	18,889	48	7,546	19	40	18,790	48	7,491	19	40	7,852	20	3,405	9	43
Any coronary hospitalization**	43,507	25,909	60	7,468	17	29	13,821	32	4,911	11	36	13,775	32	4,869	11	35	5,545	13	2,100	5	38
# of cardiologist visits	0	0		0			0		0			0		0			0		0		
# of other physician visits	3	4		3			3		3			3		3			4		3		
Number of different drugs	4	4		4			4		4			4		4			6		5		
# of lab tests performed	6	9		7			9		6			9		6			12		8		
**Vascular conditions:**																					
Hypercholesterolemia	358,649	308,261	86	100,890	28	33	286,144	80	88,027	25	31	282,875	79	87,016	24	31	74,655	21	23,200	6	31
Hypertension	351,542	244,573	70	100,813	29	41	207,406	59	85,722	24	41	205,531	58	84,903	24	41	75,677	22	33,388	9	44
Heart failure	16,008	10,057	63	3,243	20	32	5,082	32	1,917	12	38	5,064	32	1,891	12	37	2,884	18	1,089	7	38
Acute MI	20,797	11,127	54	2,673	13	24	5,074	24	1,709	8	34	5,046	24	1,698	8	34	1,966	9	714	3	36
Old MI	5,166	3,734	72	998	19	27	2,345	45	714	14	30	2,320	45	705	14	30	873	17	293	6	34
Acute coron. syndrome	63,781	40,721	64	12,776	20	31	25,407	40	9,242	14	36	25,206	40	9,155	14	36	9,328	15	3,791	6	41
TIA/stroke	16,747	9,764	58	3,086	18	32	5,731	34	2,098	13	37	5,692	34	2,080	12	37	2,255	13	845	5	37
Carotid revasc^3)^	888	598	67	193	22	32	370	42	134	15	36	369	42	135	15	37	137	15	50	6	36
PVD	8,032	5,489	68	1,868	23	34	3,241	40	1,300	16	40	3,260	41	1,284	16	39	1,515	19	642	8	42
Coronary revasc^2)^	22,575	13,317	59	3,581	16	27	7,098	31	2,377	11	33	7,069	31	2,351	10	33	2,610	12	942	4	36
Peripheral revas	987	748	76	261	26	35	388	39	171	17	44	394	40	170	17	43	204	21	90	9	44
Diabetes	111,684	89,344	80	34,137	31	38	74,576	67	28,032	25	38	73,937	66	27,693	25	37	76,413	68	26,959	24	35
Pre-diabetes	9,916	8,941	90	2,559	26	29	7,957	80	2,032	20	26	7,877	79	2,016	20	26	6,258	63	1,569	16	25
Rheumatoid arthritis	4,523	3,676	81	1,520	34	41	2,650	59	935	21	35	2,620	58	926	20	35	839	19	315	7	38
Recorded obesity	20,533	17,354	85	5,295	26	31	15,138	74	4,307	21	28	14,971	73	4,251	21	28	6,897	34	1,956	10	28
COPD	27,318	18,918	69	6,382	23	34	13,236	48	4,855	18	37	13,144	48	4,818	18	37	4,557	17	1,708	6	37
Use of oxygen tank	105	59	56	20	19	34	41	39	13	12	32	42	40	13	12	31	20	19	8	8	40
**Plan type:** Indemnity	3,942	3,391	86	807	20	24	3,149	80	696	18	22	3,132	79	698	18	22	823	21	181	5	22
HMO	202,910	109,821	54	100,426	49	91	95,306	47	92,427	46	97	94,360	47	91,683	45	97	28,471	14	30,241	15	106
Medicare Advantage	62,459	37,744	60	24,757	40	66	28,014	45	20,468	33	73	27,758	44	20,304	33	73	11,658	19	8,622	14	74
Medicare Supplemental	43,645	15,519	36	1,294	3	8	7,822	18	1,000	2	13	7,745	18	994	2	13	2,647	6	285	1	11
Other	22,274	10,323	46	4,057	18	39	8,677	39	3,486	16	40	8,593	39	3,456	16	40	2,422	11	1,153	5	48
Preferred Provider Org.	368,254	304,335	83	72,802	20	24	278,740	76	60,177	16	22	276,010	75	59,645	16	22	75,743	21	16,171	4	21
**State of residence:** 1	194,150	97,286	50	51,523	27	53	83,875	43	46,332	24	55	82,694	43	46,342	24	56	25,050	13	16,146	8	64
2	23,642	20,159	85	9,281	39	46	18,674	79	8,517	36	46	18,678	79	8,521	36	46	5,416	23	2,352	10	43
3†	5,006	3,783	76	359	7	9	3,420	68	318	6	9	3,395	68	311	6	9	923	18	85	2	9
4	64,242	53,997	84	30,197	47	56	49,458	77	27,231	42	55	48,892	76	26,855	42	55	13,198	21	7,569	12	57
5	36,365	29,200	80	4,805	13	16	25,522	70	3,635	10	14	25,903	71	3,604	10	14	6,872	19	917	3	13
6	48,045	37,348	78	8,630	18	23	32,056	67	6,583	14	21	31,752	66	6,476	13	20	8,929	19	1,723	4	19
7	13,847	11,833	85	359	3	3	10,773	78	260	2	2	10,647	77	259	2	2	2,678	19	44	0	2
8	28,450	23,108	81	6,178	22	27	20,781	73	5,309	19	26	20,627	73	5,276	19	26	5,877	21	1,461	5	25
9	8,703	7,401	85	1,089	13	15	6,789	78	875	10	13	6,772	78	886	10	13	1,812	21	243	3	13
10†	3,753	2,464	66	1,183	32	48	2,226	59	1,097	29	49	2,188	58	1,083	29	49	710	19	369	10	52
11	4,864	3,610	74	2,310	47	64	3,217	66	2,030	42	63	3,137	64	2,029	42	65	1,015	21	648	13	64
12	64,237	24,644	38	36,009	56	146	15,190	24	32,988	51	217	14,979	23	32,722	51	218	6,068	9	12,646	20	208
13	85,926	71,455	83	22,481	26	31	63,065	73	18,108	21	29	62,703	73	17,993	21	29	19,587	23	5,464	6	28
Other	44,110	35,064	79	5,628	13	16	31,850	72	4,867	11	15	31,462	71	4,839	11	15	8,949	20	1,446	3	16
14†	3,804	3,311	87	116	3	4	3,113	82	100	3	3	3,107	82	99	3	3	876	23	31	1	4
15	74,340	56,470	76	23,995	32	42	51,699	70	20,004	27	39	50,662	68	19,485	26	38	13,804	19	5,509	7	40

* Study period starts July 1, 2005, ensuring a minimum CAP of 6 months with lab test results coverage and a pre-CAP of 6 months.

** Hospitalization with primary discharge code for acute MI, ACS, PCTA, PCI, CABG.

† States with no WellPoint presence.

1) This is limited to 23 lab test results reported in our study database.

2) PCTA, PCI, CABG.

3) Carotid stent, carotid endarterectomy.

Having been hospitalized in the 30 days before the initiation of lipid-lowering treatment was negatively associated with receiving an outpatient test, likely because the relevant lab tests were performed during the hospitalization and as such do not appear as outpatient lab tests. Some patients hospitalized for acute coronary syndrome or MI may have received lipid-lowering therapy for secondary prevention without the need for a lab test. This is supported by the fact that patients with both recent MI and ACS had a lower than average proportion with at least one test performed (24% and 40% compared with an average of 68%). A code for hypercholesterolemia is frequently accompanied by an LDL test performed (80%) likely because the test ordering is accompanied with such a billing code.

Patients with Medicare Supplemental coverage (43,645) had a much lower proportion of claims for LDL tests performed (18%), and of those only 2% had results available. The lab test provider may not have included the secondary payer on the claim.

The two lab test providers that provided data to the insurer do not operate in some states; for example, the availability of lab test results in one state was as low as 2% for LDL. Such low recording would not be dependent directly on patient characteristics as it affects an entire state and is driven by factors other than health status, though clinically relevant patient characteristics have varying prevalences across states.

Some patients resided in states not primarily covered by the health plan studied, and are covered only via accounts for nationally operating businesses (e.g., if the employer is based in another state, all employees may be members of a health plan in that other state, rather than the state of residence). For these patients, the availability of LDL test results is less than 10%. One state (#12) stands out as having a small proportion of patients with an outpatient LDL test performed (24%), but a much larger proportion of patients have a result available (51%). In this state, a larger proportion of providers are under HMO capitation agreements. Within these plans, under-recording of tests performed may be the result of bundled payment arrangements; however, results are still forwarded by the lab test providers resulting in the paradox of having more lab test results available in our database than performed as recorded in claims data. Among patients with Diabetes or RA, we found fundamentally similar results. Among elderly patients lab test results were more likely to be available among Medicare advantage enrollees than those patients covered through Medicare supplemental insurance (Table 4).

**Table 4:** Cross-tabulation between states and Medicare Advantage and Medicare Supplement status

	**Patients with at least 1 of any 23 lab tests**^**1)**^														
	**Patients in HMO or PPO**					**Patients in Medicare Advantage**					**Patients in Medicare Supplemental**				
**State**	**Tests performed**		**Results available**			**Tests performed**		**Results available**			**Tests performed**		**Results available**		
	**N**	**% of total**	**N**	**% of total**	**% of done**	**N**	**% of total**	**N**	**% of total**	**% of done**	**N**	**% of total**	**N**	**% of total**	**% of done**
1	87,110	52	48,838	29	56	3,999	51	1,978	25	49	5,881	31	625	3	11
2	17,068	89	8,331	43	49	174	85	95	46	55	574	37	92	6	16
3†	3,507	81	329	8	9	16	44	12	33	75	141	39	3	1	2
4	53,350	86	30,037	48	56	112	84	64	48	57	519	26	92	5	18
5	26,240	84	4,454	14	17	1,036	80	167	13	16	999	38	55	2	6
6	30,291	86	7,265	21	24	2,850	79	1,139	32	40	4,012	45	185	2	5
7	11,050	89	346	3	3	0	0	1	100	0	564	47	6	1	1
8	22,277	85	6,068	23	27	4	14	7	24	175	692	34	71	3	10
9	6,449	89	1,003	14	16	0	0	0	0	0	278	40	8	1	3
10†	2,372	66	1,144	32	48	3	23	5	38	167	5	31	0	0	0
11	3,547	76	2,289	49	65	1	33	2	67	200	38	21	9	5	24
12	15,645	43	19,387	54	124	6,560	30	14,189	65	216	105	39	15	6	14
13	46,115	86	15,049	28	33	22,884	84	7,035	26	31	1,543	39	126	3	8
Other	32,651	81	5,515	14	17	54	39	54	39	100	153	37	5	1	3
14†	3,256	88	113	3	3	0	0	0	0	0	7	30	0	0	0
15	53,228	82	23,060	36	43	51	59	9	10	18	8	33	2	8	25

† States with no WellPoint presence.

**Figure 3:**
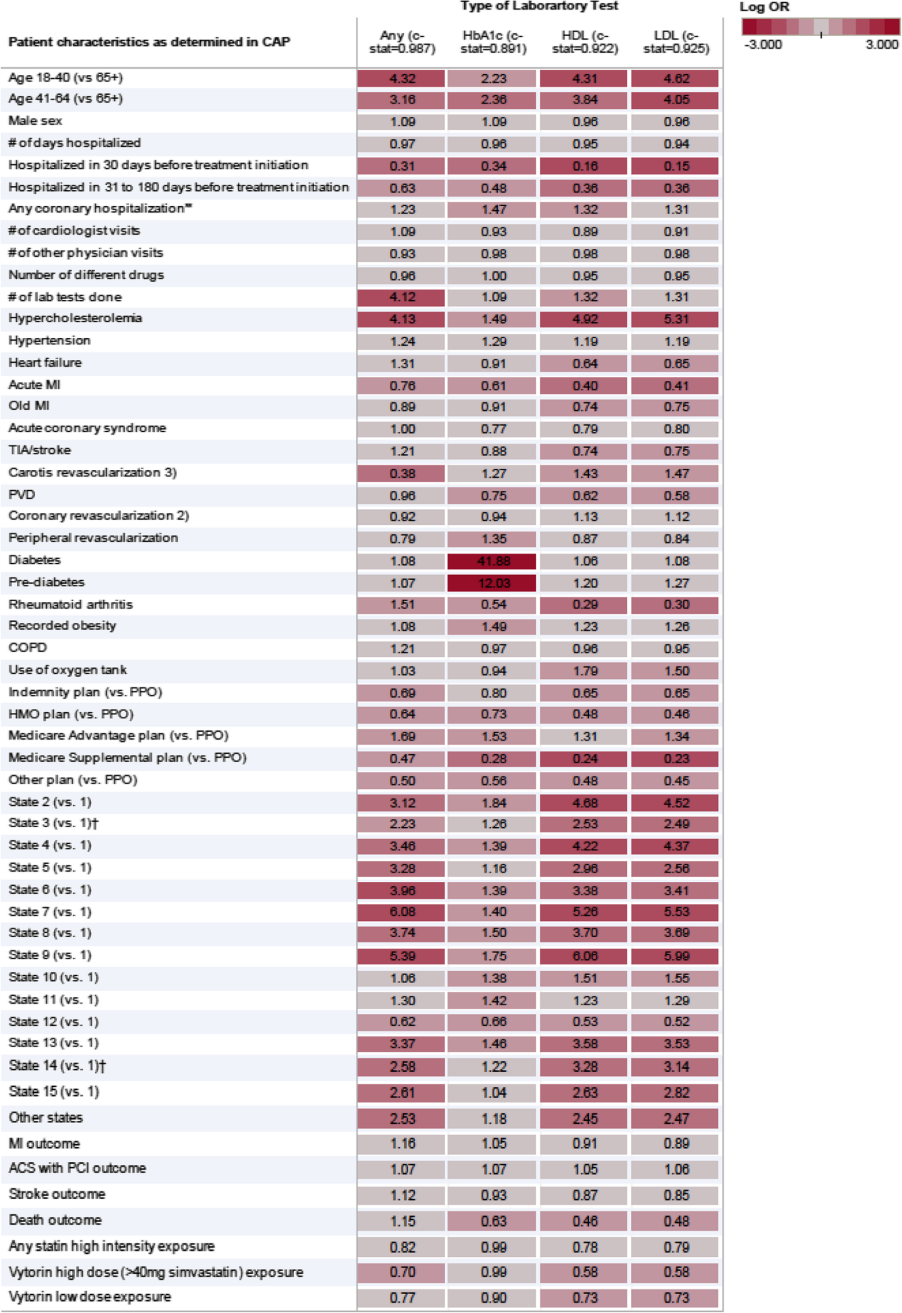
Associates of selected outpatient lab tests performed in patients initiating lipid-lowering treatment according to claims data in 703,484 patients from a logistic regression model (darker means stronger association)

Based on patient and system characteristics plus exposure and outcome status it was possible to predict with high sensitivity (97%) and specificity (94%) whether outpatient lab tests were performed in the 6 months before treatment initiation. The corresponding model c-statistics of the logistic regression models were between 0.89 and 0.93 (Figure 3), indicating a very high predictive capacity. Strong independent associates of having an outpatient LDL test performed were a diagnosis of hypercholesterolemia or obesity, and carotid revascularization. Associates of low probability of doing LDL lab tests were recent hospitalization and being diagnosed with RA. Being older than 65 also decreased the chance of an LDL lab test, likely because of test underreporting due to bundled payments. Initiating high-intensity lipid-lowering treatment and dying in the study follow-up period were correlates of not having an outpatient LDL, HDL, or HB_A1c_ test performed. Not surprisingly, the strongest predictor of having an HB_A1c_ test performed was a diagnosis of diabetes or pre-diabetes.

**Figure 4:**
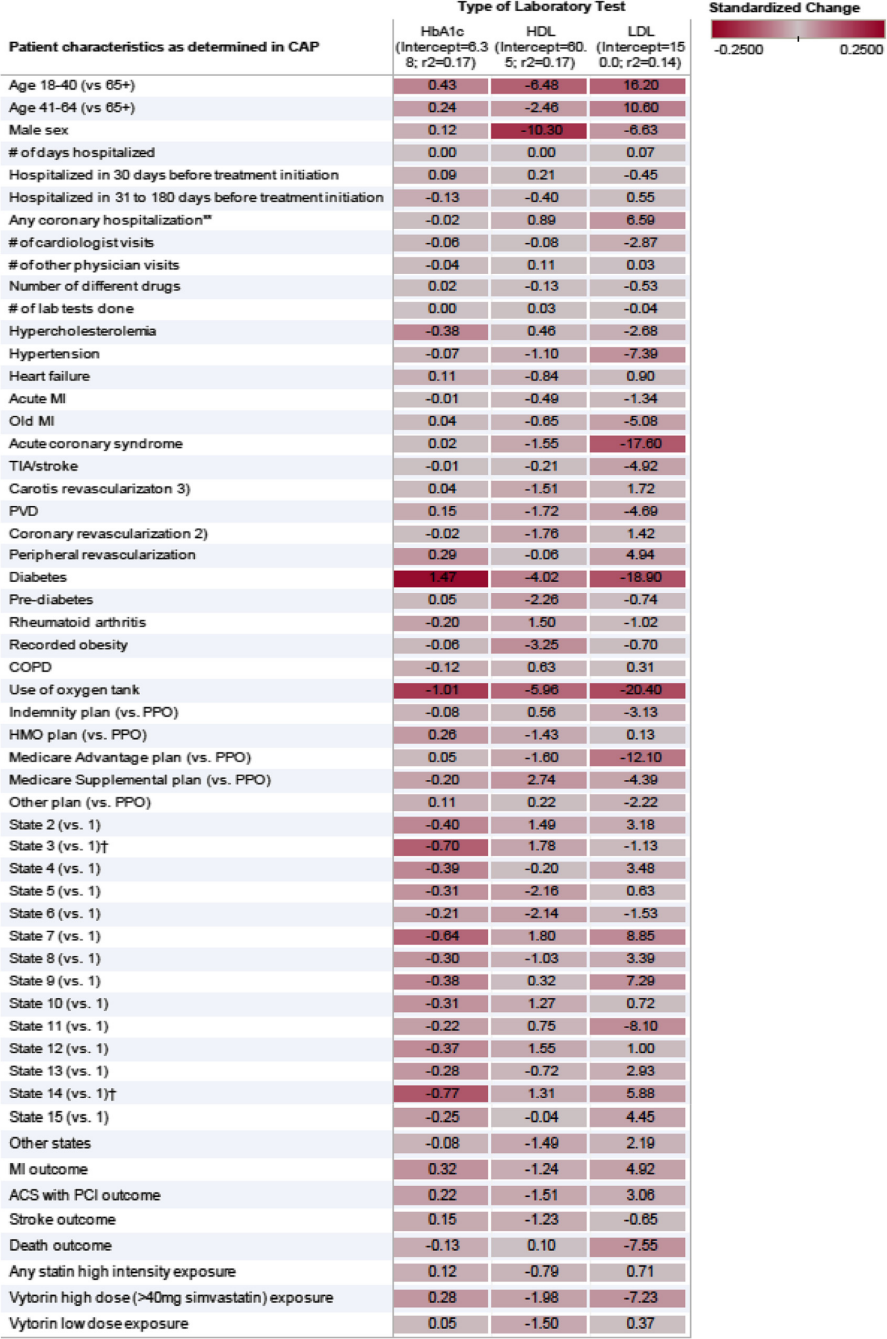
Correlates of selected lab test results among patients with lab test results available (darker means stronger correlations)

Among the patients for whom LDL, HDL, or HB_A1c_ test levels were available, we then attempted to predict the actual lab levels based on their recorded patient and system characteristics. Using all observed factors described above, 17% of the variation could be explained (Figure 4). Young age was the strongest correlate of increased high LDL (+20 mg/dl) and Hb_A1c_ (+0.5%) levels, suggesting that in younger age initiation of lipid-lowering therapy was more driven by lab test results, i.e. primary prevention, while in older age past coronary events and other risk factors were the triggers for statin initiation despite lower LDL levels (−17 mg/dl).

Higher intensity of lipid lowering treatment generally was correlated with a lower proportion of outpatient LDL tests performed, a lower fraction of LDL test results available in the database, and lower LDL serum levels (Table 5). For example, among high dose simvastatin initiators (>40 mg/day), 52% had an outpatient LDL test performed before treatment start (63% for lower dose simvastatin). Of those patients, 37% had a test result available (42%), and the mean LDL serum level was 135.6 mg/dl compared to 147.3 mg/dl for patients started on low-intensity simvastatin. Mean LDL levels were generally lower in patients with diabetes who initiated lipid-lowering therapy.

**Table 5:** LDL tests performed and LDL test results available by lipid lowering treatment in patients initiating lipid-lowering therapy, including patient subgroups with diabetes (DM) or rheumatoid arthritis (RA)

		**All patients**		**LDL test performed**		**LDL test results available**			**LDL level**		**HDL level**	
	**Initiated medication**	**N**	**%**	**N**	**%**	**N**	**% of total N**	**% of ordered**	**mean**	**s.d.**	**mean**	**s.d.**
All	Any statin alone	655,211	93	395,496	60.4	165,380	25.2	41.8	145.3	38.6	50.2	14.5
	Any statin high intensity*	173,979	25	98,309	56.5	41,320	23.7	42.0	144.2	45.5	49.2	14.3
	Any statin low intensity	481,232	68	297,187	61.8	124,060	25.8	41.7	145.7	36	50.6	14.6
	Simvastatin alone	282,658	40	176,697	62.5	73,193	25.9	41.4	147.0	36.3	50.2	14.4
	Simvastatin high dose (>40 mg)	11,354	2	5,912	52.1	2,192	19.3	37.1	135.6	49.5	48	13.6
	Simvastatin low dose	271,304	39	170,785	62.9	71,001	26.2	41.6	147.3	35.8	50.2	14.4
	Vytorin alone	24,931	4	12,966	52	6,820	27.4	52.6	142.2	47.6	49.6	14.3
	Vytorin high dose (>40 mg simvast)	1,069	0	563	52.7	223	20.9	39.6	124.1	56.7	48.1	13.5
	Vytorin low dose	23,862	3	12,403	52	6,597	27.6	53.2	142.8	47.1	49.6	14.4
	Ezetimibe alone	20,509	3	11,412	55.6	5,334	26	46.7	143.2	38.1	51.5	14.9
	Ezetimibe + statin	2,833	0	1,834	64.7	720	25.4	39.3	145.7	47.7	48.2	13.5
	**Total:**	**703,484**										
DM	Any statin alone	103,664	93	69,788	67.3	25,927	25	37.2	124.8	37.4	46.5	13.1
	Any statin high intensity*	28,472	26	18,069	63.5	6,909	24.3	38.2	122.3	43.3	45.8	13.1
	Any statin low intensity	75,192	67	51,719	68.8	19,018	25.3	36.8	125.7	35.0	46.8	13.1
	Simvastatin alone	46,414	42	32,207	69.4	11,670	25.1	36.2	126.8	35.6	46.5	13.0
	Simvastatin high dose (>40 mg)	2,498	2	1,513	60.6	521	20.9	34.4	117.1	46.2	45.6	12.6
	Simvastatin low dose	43,916	39	30,694	69.9	11,149	25.4	36.3	127.2	35.0	46.6	13.0
	Vytorin alone	4,049	4	2,324	57.4	1,086	26.8	46.7	122.9	44.4	45.6	13.4
	Vytorin high dose (>40 mg simvast)	269	0	169	62.8	58	21.6	34.3	105.6	41.6	46.3	14.0
	Vytorin low dose	3,780	3	2,155	57	1,028	27.2	47.7	123.8	44.4	45.6	13.4
	Ezetimibe alone	3,459	3	2,092	60.5	884	25.6	42.3	126.9	37.7	47.0	13.1
	Ezetimibe + statin	512	1	372	72.7	135	26.4	36.3	133.1	44.5	44.8	11.8
	**Total:**	**111,684**										
RA	Any statin alone	4,118	91	2,425	58.9	833	20.2	34.4	138.5	39.4	54.7	16.5
	Any statin high intensity*	1,044	23	575	55.1	205	19.6	35.7	135.4	50.9	53.6	17.8
	Any statin low intensity	3,074	68	1,850	60.2	628	20.4	33.9	139.5	34.8	55.1	16.0
	Simvastatin alone	1,749	39	1,070	61.2	360	20.6	33.6	141.6	33.4	54.3	15.5
	Simvastatin high dose (>40 mg)	70	2	37	52.9	14	20	37.8	134.6	44.2	46.5	12.0
	Simvastatin low dose	1,679	37	1,033	61.5	346	20.6	33.5	141.9	32.9	54.6	15.6
	Vytorin alone	174	4	85	48.9	43	24.7	50.6	142.6	45.6	52.8	16.3
	Vytorin high dose (>40 mg simvast)	9	0	4	44.4	2	22.2	50.0	177.0	76.4	52	22.6
	Vytorin low dose	165	4	81	49.1	41	24.8	50.6	141.0	44.5	52.8	16.3
	Ezetimibe alone	210	5	126	60	55	26.2	43.7	137.5	31.3	56.8	16.7
	Ezetimibe + statin	21	1	14	66.7	4	19	28.6	144.0	45.5	49.0	10.4
	**Total:**	**4,523**										

* See methods section for definition.

## Discussion

We studied the characteristics of laboratory test information in a pharmacoepidemiologic research data source that enriches longitudinal claims data with outpatient lab test results data, which makes it possible to better adjust for biomarkers of cardiac risk in comparative effectiveness studies. In an example cohort study of 703,484 patients initiating various lipid-lowering therapies, 68% of patients had at least one of a set of 23 study lab tests performed in the 6 months before treatment, and 42% of those had test results available. LDL test results were available for 24% of statin initiators, a non-trivial level of missingness that needed to be addressed in order to preserve the validity and generalizability of findings. Missingness due to absence of lab tests being performed followed a complex pattern that is largely explained by hospitalization, clinical practice guidelines which differ for primary and secondary prevention of coronary heart disease, and by some health care system characteristics.

Several key points regarding these patterns arose and have implications for conducting comparative effectiveness research studies in such enriched data sources.

### Operational aspects

A covariate assessment period of 6 months was sufficient to capture the majority of outpatient lab tests performed. Extending the period to 9 and 12 months, and thus extending the required pre-exposure enrollment period, provided few additional observed lab tests but may disproportionally reduce the cohort size if working with health plans that have high enrollee turnover rates. Patients with existing chronic conditions like RA or diabetes have more outpatient lab tests available if their healthcare providers monitor them more closely. Recent hospitalizations strongly decreased the number of outpatient lab tests. It is likely that tests were performed during the hospitalization, and if the test results were available to the patient's primary care physician, repeating testing may not have been required for some time after discharge.

### Selectiveness of lab tests performed

Patients with risk factors for cardiovascular events were less likely to have lab tests performed. Many patients with these characteristics receive lipid-lowering treatment as secondary prevention, which is initiated independent of serum lipid-levels more frequently than is primary prevention. Indeed, treatment guidelines in place since the late 1990s recommend that patients with a major cardiac event should be treated with lipid lowering medications [15,16]. In a prior study, patients who initiated high-intensity lipid-lowering treatment were less likely to have had an outpatient lab test performed [17]. Because the presence of preexisting cardiac risk factors is both a strong predictor of future events and a predictor of missing data on lipid levels, disregarding the missing information can be expected to bias findings of non-randomized comparative effectiveness research in this setting.

### Selectiveness of lipid lab test results available

Among patients who had lab test result available, those who were subsequently initiated on higher-intensity lipid-lowering treatment were more likely to have lower lipid serum levels. This finding is again compatible with clinical practice and trial findings that patients with acute coronary events (who are less likely to have outpatient lipid tests available) should be treated with high-intensity statins largely independent of their lipid levels [17,18]. System factors like state of residence and insurance plan type, particularly supplemental insurance, may substantially influence the availability of test results. However, since those factors are less likely to be systematically related to health outcomes it is unlikely that these will act as major confounding factors in comparative effectiveness studies.

In addition to these limitations regarding lab tests, baseline clinical conditions may be under-reported through claims data in some patients, particularly when using a short ascertainment period, such as the 6-month period we used. For LDL, HDL, and HB_A1c_ tests it is unlikely that point-of-care testing would be performed, which the lab test provider chain would not record. However, other tests, like INR, urine analyses and creatinine levels might be subject to this additional limitation.

**Figure 5:**
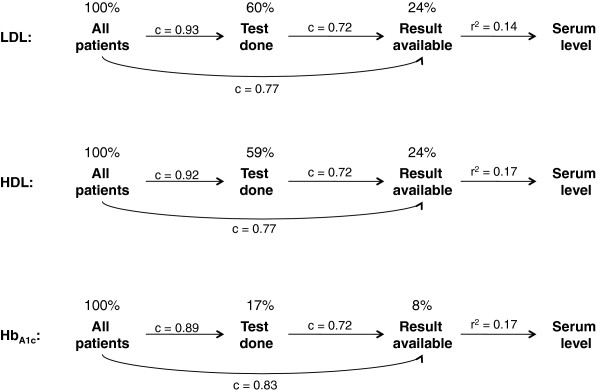
Ability of longitudinal claims data to predict whether a lab test was performed, a test result was available, and the actual serum level for three biomarkers of cardiovascular risk*. * c-statistics were computed from multivariate logistic regression models including patient factors measured during 6 months before lipid-lowering treatment initiation; r^2^ measured were computed only among patients who had a lab test result available from linear regression including patient factors measured during 6 months before lipid-lowering treatment initiation, actual treatment choice, as well as cardiovascular events and death during follow-up

If replicated in other patient populations and datasets, these findings have important implications for CER studies. The complexity of the nature of missingness that logically follows from clinical practice and the reality of our health care system requires the inclusion of a wide variety of patient and system characteristics in order to model the missing data structure. In our specific example the combination of primary and secondary prevention with lipid-lowering medications seems to complicate the prediction of missing values, but in the end is likely a reason why we could differentiate so well between patients who have an outpatient LDL test performed versus not (Figure 5). Once the outpatient lab test results were available, we had moderate ability to predict the exact lipid/Hb_A1c_ serum level. The resulting mismeasurement of imputed lab test results suggests that imputation of test results would provide only limited additional confounding control. However, estimation precision would be increased because the analyzable population would more than triple in our example study.

It is likely that the specific patterns of missingness of outpatient lab test results will vary depending on the clinical scenario, health care practice, and system constraints. It is encouraging that despite the non-random missingness we were able to predict quite well who would and would not receive a lab test result, which is a good starting point for addressing this issue. However, our difficulty in predicting actual lab values is a challenge to incorporating lab data through imputation or weighting approaches in comparative effectiveness research studies.

## Conclusion

In a claims database linked with outpatient lab test results, we found that lab tests are performed selectively depending on patient risk factors and corresponding to current treatment guidelines. Poor ability to predict lab values and the high proportion of missingness reduces the added value of lab tests for effectiveness research in this setting.

## Competing interests

Dr. Schneeweiss is Principal Investigator of the Brigham and Women’s Hospital DEcIDE Center on Comparative Effectiveness Research and the DEcIDE Methods Center both funded by AHRQ and of the Harvard-Brigham Drug Safety and Risk Management Research Center funded by FDA. Dr. Schneeweiss is paid consultant to WHISCON LLC and Booz & Co, and he is principal investigator of investigator-initiated grants to the Brigham and Women’s Hospital from Pfizer, Novartis, and Boehringer-Ingelheim unrelated to the topic of this study.

## Authors’ contributions

SS, and JAR conceived the idea through their interests in improving confounding adjustment by adding information from electronic medical records to claims databases. All authors supported the design, analysis, and interpretation in various ways. All authors critically reviewed the drafts and approved the final version.

## Disclosures

Dr. Schneeweiss is Principal Investigator of the Brigham and Women’s Hospital DEcIDE Center on Comparative Effectiveness Research and the DEcIDE Methods Center both funded by AHRQ and of the Harvard-Brigham Drug Safety and Risk Management Research Center funded by FDA. Dr. Schneeweiss is paid consultant to WHISCON and Booz & Co, and he is principal investigator of investigator initiated grants to the Brigham and Women’s Hospital from Pfizer, Novartis, and Boehringer-Ingelheim. Drs. Daniel was and Singer is employed by HealthCore, a subsidiary of WellPoint.

## Pre-publication history

The pre-publication history for this paper can be accessed here:

http://www.biomedcentral.com/1471-2288/12/180/prepub

## Supplementary Material

Additional file 1 **Appendix Table S1.** Lab test results available in the linked study database. **Table S2.** Clinical covariate definitions. **Table S3.** Outcome definitions. Click here for file

## References

[B1] Schneeweiss S Avorn J A review of uses of health care utilization databases for epidemiologic research on therapeutics J Clin Epidemiol 2005 58 323 337 10.1016/j.jclinepi.2004.10.012 15862718

[B2] Seeger JD Walker AM Williams PL Saperia GM Sacks FM A propensity score-matched cohort study of the effect of statins, mainly fluvastatin, on the occurrence of acute myocardial infarction Am J Cardiol 2003 92 1447 1451 10.1016/j.amjcard.2003.08.057 14675584

[B3] Mulla ZD Seo B Kalamegham R Nuwayhid BS Multiple imputation for missing laboratory data: an example from infectious disease epidemiology Ann Epidemiol 2009 19 908 914 10.1016/j.annepidem.2009.08.002 19811933

[B4] Greenland S Finkle WD A critical look at methods for handling missing covariates in epidemiologic regression analyses Am J Epidemiol 1995 142 1255 1264 7503045 10.1093/oxfordjournals.aje.a117592

[B5] Vach W Blettner M Biased estimation of the odds ratio in case–control studies due to the use of ad hoc methods of correcting for missing values for confounding variables Am J Epidemiology 1991 134 895 907 10.1093/oxfordjournals.aje.a116164 1670320

[B6] Neri L Rocca Rey LA Lentine KL Joint association of hyperuricemia and reduced GFR on cardiovascular morbidity: a historical cohort study based on laboratory and claims data from a national insurance provider Am J Kidney Dis 2011 58 398 408 10.1053/j.ajkd.2011.04.025 21783292

[B7] Laitinen DL Manthena S Impact of change in high-density lipoprotein cholesterol from baseline on risk for major cardiovascular events Adv Ther 2010 27 233 244 10.1007/s12325-010-0019-4 20437214

[B8] McCullough E Sullivan C Banning P Goldfield N Hughes J Challenges and benefits of adding laboratory data to a mortality risk adjustment method Qual Manag Health Care 2011 20 253 262 21971023 10.1097/QMH.0b013e318231cf4f

[B9] Schneeweiss S A basic study design for expedited safety signal evaluation based on electronic healthcare data Pharmacoepidemiol Drug Saf 2010 19 858 868 10.1002/pds.1926 20681003 PMC2917262

[B10] Choudhry NK Levin R Winkelmayer WC Statins in elderly patients with acute coronary syndrome: an analysis of dose and class effects in typical practice Heart 2007 93 945 951 10.1136/hrt.2006.110197 17344334 PMC1994395

[B11] Schneeweiss S Seeger JD Maclure M Wang PS Avorn J Glynn RJ Performance of comorbidity scores to control for confounding in epidemiologic studies using claims data Am J Epidemiol 2001 154 854 864 10.1093/aje/154.9.854 11682368

[B12] Moons KG Donders RA Stijnen T Harrell FE Jr Using the outcome for imputation of missing predictor values was preferred J Clin Epidemiol 2006 59 1092 1101 10.1016/j.jclinepi.2006.01.009 16980150

[B13] Kiyota Y Schneeweiss S Glynn RJ Cannuscio CC Avorn J Solomon DH Accuracy of Medicare claims-based diagnosis of acute myocardial infarction: estimating positive predictive value on the basis of review of hospital records Am Heart J 2004 148 99 104 10.1016/j.ahj.2004.02.013 15215798

[B14] Kannel WB Wilson PW Efficacy of lipid profiles in prediction of coronary disease Am Heart J 1992 124 768 774 10.1016/0002-8703(92)90288-7 1514505

[B15] Braunwald E Antman EM Beasley JW ACC/AHA guidelines for the management of patients with unstable angina and non-ST-segment elevation myocardial infarction: executive summary and recommendations. A report of the American College of Cardiology/American Heart Association task force on practice guidelines (committee on the management of patients with unstable angina) Circulation 2000 102 1193 1209 10.1161/01.CIR.102.10.1193 10973852

[B16] Kushner FG Hand M Smith SC Jr 2009 Focused Updates: ACC/AHA Guidelines for the Management of Patients With ST-Elevation Myocardial Infarction (updating the 2004 Guideline and 2007 Focused Update) and ACC/AHA/SCAI Guidelines on Percutaneous Coronary Intervention (updating the 2005 Guideline and 2007 Focused Update): a report of the American College of Cardiology Foundation/American Heart Association Task Force on Practice Guidelines Circulation 2009 120 2271 2306 10.1161/CIRCULATIONAHA.109.192663 19923169

[B17] Cannon CP Braunwald E McCabe CH Intensive versus moderate lipid lowering with statins after acute coronary syndromes N Engl J Med 2004 350 1495 1504 10.1056/NEJMoa040583 15007110

[B18] de Lemos JA Blazing MA Wiviott SD Early intensive vs a delayed conservative simvastatin strategy in patients with acute coronary syndromes: phase Z of the A to Z trial JAMA 2004 292 1307 1316 10.1001/jama.292.11.1307 15337732

