# Supplementary material for: Supplementing claims data with outpatient laboratory test results to improve confounding adjustment in effectiveness studies of lipid-lowering treatments
Source: BMC Med Res Methodol. 2012 Nov 26;12:180. doi: 10.1186/1471-2288-12-180 (PMC3533513; doi:10.1186/1471-2288-12-180)
Supplement: Additional file 1 — Appendix Table S1. Lab test results available in the linked study database. Table S2. Clinical covariate definitions. Table S3. Outcome definitions. [file 1471-2288-12-180-S1.doc]

Appendix

Definitions of covariates, lab test results and study endpoints

Clinical covariates were defined using diagnostic and procedure information (Table 1). Lab test results were defined using LOINC codes (Table 2) and outcomes were defined using combinations of diagnostic and procedure information (Table 3).

**Table S1: Clinical Covariate Definitions**

We consider the 6 months immediately preceding and including the drug initiation date as the covariate assessment period

| **Variable** | **Coding** |
| --- | --- |
| **Vascular conditions & risk factors:** |  |
| Hypercholesterolemia | 1 in- or out-patient claim with one of ICD9 diagnosis codes 272.0, 272.2, 272.4 |
| Hypertension | At least 1 diagnosis (ICD9 401.x- 405.x) or one dispensing of a CCB, ACE, ARB, TZD, BB, or a direct antihypertensive agent. |
| Heart failure | 1 inpatient or 2 outpatient claims with any of ICD9 diagnosis codes: 428.x, 398.91, 402,01, 402.11, 402.91, 404.01, 404.11, 404.91, 404.03, 404.13, 404.93 |
| Acute MI | 1 in- or out-patient claim with ICD-9 diagnosis code 410.x |
| Old MI | 1 in- or out-patient claim with ICD-9 diagnosis code 412.x |
| Acute coronary syndrome | 1 inpatient or 2 outpatient claims with any of ICD9 diagnosis codes 410.x-414.x |
| TIA/stroke | 1 inpatient or 2 outpatient claims with any of the following ICD9 diagnosis codes: 430.x – 433.x, 434.x1, 435.x, 436.x, 437.1, 438.x |
| Carotid revascularization | 1 inpatient or 2 outpatient claims with any of the following codes: |
|  | ICD9 procedure: |
|  | 00.63, 00.61, 38.12 |
|  | HCPCs: |
|  | 0075 T, 34001, 35301, 35694, 35695, 35501, 35506, 35508, 35509, 35601, 35606 |
| Peripheral vascular disease | 1 inpatient or 2 outpatient claims with any of the following codes: |
|  | ICD9 diagnosis: |
|  | 440.20 - 440.24, 440.29 – 440.32, 440.3, 443.9 |
|  | ICD9 procedure: |
|  | 38.08, 38.09, 38.18, 38.48, 38.49, 39.25, 39.5, 39.9, 84.10 - 84.17 |
|  | HCPCs: |
|  | 35256, 35286, 35351, 35355, 35361, 35363, 35371, 35372, 35381, 35454, 35456, 35459, 35470, 35473, 35474, 35482, 35483, 35485, 35492, 35493, 35495, 35521, 35533, 35541, 35546, 35548, 35549, 35551, 35556, 35558, 35563, 35565, 35566, 35571, 35621, 35623, 35641, 35646, 35647, 35650, 35651, 35654, 35656, 35661, 35663, 35666, 35671, 27590, 27591, 27592, 27594, 27596, 27880, 27881, 27882, 27884, 27886, 27888 |
| Coronary revascularization | 1 inpatient or 2 outpatient claims with any of the following codes: |
|  | ICD9 procedure: |
|  | 36.01, 36.02, 36.05, 36.06, 36.07,36.09,36.1, 36.2 |
|  | HCPCs: |
|  | 33510–33536 |
|  | 33545, 33572 |
| Peripheral revascularization | 1 inpatient or 2 outpatient claims with any of the following codes: |
|  | ICD9 procedure: |
|  | 39.25, 39.29, 39.90, 00.55 |
|  | HCPCs: |
|  | 34201, 34203, 35355,35361, 35363, 35371, 35372, 35456 |
|  | 35302–35306 |
|  | 35539–35566 |
|  | 35583–35587 |
|  | 35651–35671 |
|  | 35474–35476 |
|  | 35480–35485 |
|  | 35492 - 35495 |
| Diabetes | At least 2 outpatient diagnoses of DM (ICD9 250.x) in past 6 months or 1 hospital discharge diagnosis of DM in past 6 months or 1 diagnosis of DM plus an insulin or oral antidiabetic dispensing |
| Pre-diabetes | 1 inpatient or 1 outpatient claim with ICD9 diagnosis code 790.29 |
| Rheumatoid arthritis | At least 2 outpatient diagnoses of RA (ICD9 714.x) in past 6 months or 1 hospital discharge diagnosis of RA in past 6 months or 1 diagnosis of RA plus a DMARD drug dispensing |
| Recorded obesity | 1 in- or out-patient claim with ICD-9 278.0x |
| COPD (Chronic bronchitis or emphysema) | 1 in- or out-patient claim with ICD-9 diagnosis codes 491.x, 492.x, 496.x |
| Oxygen canister use | 1 in- or out-patient claim with ICD-9 procedure: 93.96 (Oxygen therapy) |

**Table S2: Lab test results available in the linked study database**

|  | **Test Description** | **Logical Observation Identifiers Names and Codes (LOINC) used to identify lab test result** | **CPT-4 and ICD-9 procedure codes used to identify lab tests performed** |
| --- | --- | --- | --- |
| 1 | LDL - direct & calculated | 13457-7, 18262–6, 2089–1, 2090–9, 2532-0 | 83721, 83701, 83704, 80061 |
| 2 | HDL | 2085-9, 2086-7 | 83718, 80061 |
| 3 | Total Cholesterol | 2093-3 | 82465, 80061 |
| 4 | Triglycerides | 2571-8, 3049-4 | 84478, 80061 |
| 5 | Apolipoprotein A1 | 1869-7 | 82172 |
| 6 | Apolipoprotein B | 1871-3, 1884-6 | 82172 |
| 7 | Hemoglobin A1c | 4548-4 | 83036 |
| 8 | Hemoglobin | 18310-3, 5794–3, 718-7 | 85018, 85025, 85027, 85032 |
| 9 | White Blood Cell Count | 6690-2, 806-0 | 85048, 85025, 85027, 85032 |
| 10 | Differential Cell Counts | 19023-1, 23761–0, 26505–8, 26511–6, 768–2, 769–0, 770–8, 26478–8, 731–0, 736–9, 737–7, 26485–3, 5905–5, 744–3, 30180–4, 704–7, 706–2, 707–0, 26450–7, 711–2, 713–8, 714-6 | 85048, 89051, 85025 |
| 11 | INR (International Normalized Ratio) | 5894-1, 6301-6 | 85610 |
| 12 | ALT Alanine Transaminase (SGPT) | 1742-6 1743-4 | 84460, 80053, 80054, 80076, 80058 |
| 13 | AST Aspartate Transaminase (SGOT) | 1920-8 30239-8 | 84450, 80053, 80054, 80076, 80058 |
| 14 | Uric Acid | 16259-4, 3084–1, 3085–8, 32150-5 | 84550 |
| 15 | Serum Creatinine | 2160-0 | 84520 |
| 16 | Microalbumin UR Qn | 14957-5 | 82043 |
| 17 | Microalbumin/creat Ur-mRto | 14959-1 | 82043 |
| 18 | High Sensitivity CRP | 1988-5 30522-7 | 86141, 86140 |
| 19 | Cyclic citrullinated peptide, antibody (CCP) | 33935-8 | 86200 |
| 20 | Rheumatoid Factor (RF or RhF) | 11572-5 11573–3 15205–8 33313–8 33314–6 33648–7 5299-3 | 86430, 86431 |
| 21 | Hepatitis B Surface Antigen (HBsAg or HBeAg) | 47364-5 5195–3 5196–1, 13954–3 31844–4 5191–2 9589-3 | 87340, 87341, 80074, 88618, 87350 |
| 22 | Hepatitis B DNA (HB DNA) Qualitative / Quantitative | 29610-3 5007–0 5009–6, 11258–1 29615–2 42595-9 | 87516, 87517 |
| 23 | ESR Erythrocyte sedimentation rate | 4537-7, 4538-5 | 85651, 85652 |

**Table S3: Outcome definitions**

| **Outcome** | **Definition** |
| --- | --- |
| Myocardial infarction | Hospitalization for MI |
| Stroke | Hospitalization for a cerebrovascular event excluding TIA |
| ACS with PCI | Hospitalization for acute coronary syndrome with a revascularization procedure |
| Death | Death of any cause |

Coding of Myocardial infarction

Codes: ICD-9-CM from Part A (inpatient): 410.01, 410.11, 410.21, 410.31, 410.41, 410.51, 410.61, 410.71, 410.81, 410.91; DRG 121, 122, 123

Algorithm: Hospitalization episode lasting at least 3 days and no more than 180 days with one of the following ICD-9-CM diagnosis codes: 410.01, 410.11, 410.21, 410.31, 410.41, 410.51, 410.61, 410.71, 410.81, or 410.91. These diagnosis codes must have been listed on the discharge abstract as the principal reason for admission (principal diagnosis) or the next diagnosis (secondary diagnosis). Re-admissions for AMI (410 with a fifth digit of 2 indicating the prior hospitalization due to AMI within 8 weeks) were excluded, to identify only initial hospitalizations. The length of stay could have been less than three days if the subject died during the hospitalization.

Coding of Cerebrovascular Events

430.x Subarachanoid hemorrhage

431.x Intracerebral hemorrhage

432.x Other and unspecified intracranial hemorrhage

433.x Occlusion and stenosis of precerebral arteries

434.x Occlusion of cerebral arteries

436.x Acute but ill-defined cerebrovascular disease

437.x Other and ill-defined cerebrovascular disease

Coding of acute coronary syndrome (ACS) with revascularization

Any diagnosis code of 410.xx – 414.xx (where x can be any or no 4th / 5th digit) and ICD-9 and CPT-4 procedure codes for PTCA, any coronary stenting, CABG surgery:

PTCA:

CPT-4: 92982, 92995, 92997, 92982–92984 or ICD-9 proc: 00.66, 36.03, 36.09

or DRG: 112, 555

Stenting:

CPT-4 92980, 92981 or ICD-9 procedure: 36.06, 36.07 or DRG: 556, 557,558

CABG:

CPT-4: 33510 – 33545 or ICD-9 procedure: 36.1x, 36.2x or DRG: 106, 107, 109, 547, 548, 549, 550
